# Advanced Nasopharyngeal Carcinoma with Generalized Lymphadenopathy Masquerade as Lymphoma

**DOI:** 10.22038/IJORL.2022.62088.3133

**Published:** 2022-05

**Authors:** V Sha Kri Eh Dam, Nasibah Mohamad, Izyan Rifhana Muhamad, Al Duais Saleh Abdul Kadir Saeed, Wan Faiziah Wan Abdul Rahman, Faezahtul Arbaeyah Hussain, Azlan Husin, Norhafiza Mat Lazim

**Affiliations:** 1 *Department of Otorhinolaryngology-Head & Neck Surgery, School of Medical Sciences, University Sains Malaysia Health Campus, 16150 Kota Bharu, Kelantan, Malaysia. *; 2 *Department of Radiology, School of Medical Sciences, University Sains Malaysia, Health Campus, 16150 Kota Bharu, Kelantan, Malaysia.*; 3 *Department of Pathology, School of Medical Sciences, University Sains Malaysia, Health Campus, 16150 Kota Bharu, Kelantan, Malaysia.*; 4 *Department of Internal Medicine, School of Medical Sciences, University Sains Malaysia, Health Campus, 16150 Kota Bharu, Kelantan, Malaysia.*

**Keywords:** Generalized lymphadenopathy, Nasopharyngeal carcinoma, Neck metastases, lymphoma

## Abstract

**Introduction:**

Nasopharyngeal carcinoma (NPC) is a nasopharyngeal epithelial neoplasm that has distinct aetiological, epidemiological and biological characteristics compared to other head and neck malignancies. Patients usually present late due to non-specific symptoms and deep location of the tumour in the nasopharynx.

**Case Report:**

We would like to highlight a case of advanced NPC presenting with generalised lymphadenopathy, without the presence of an obvious nasopharyngeal mass that masqueraded as lymphoma in the initial stage.

**Conclusions:**

NPC may share clinical features with other sinonasal pathologies or other malignant lymphoproliferative disorders that lead to a delay in diagnosis. NPC should be one of the differential diagnoses for any cases presenting with cervical lymphadenopathy, especially in adult male patients originating from East or Southeast Asia. Early diagnosis and treatment are crucial because early-stage NPC has an excellent chemoradiotherapy response and high survival rate.

## Introduction

Nasopharyngeal carcinoma (NPC) is an epithelial carcinoma arising from the nasopharyngeal epithelium. It has unique aetiological, epidemiological and biological characteristics that differentiate it from other head and neck malignancies (1). In addition, the deep location of the nasopharynx, specifically at the fossa of Rosenmuller (FOR), where the tumour commonly originates, makes the early detection of the disease difficult. It is a relatively rare tumour globally, accounting for only 0.7% of all cancers diagnosed in 2018 and being the 23rd most common cancer in the world (2,3). However, it is not uniformly distributed geographically, with 85% of all cases from Asia (3). East Asia, especially China and Southeast Asia, are among the countries that significantly contribute to the high proportion (1,3).

NPC is more common in males, with a male-to-female ratio of 3:1, and the middle-aged population is the most commonly affected group in endemic areas (1,4). Neck swelling is the most common presenting symptom, followed by nasal and ear symptoms (1,4,5). Neurological and systemic metastasis presentations are rare but denote advanced disease. Although cervical lymph node (LN) metastasis is widespread, generalised involvement of LN elsewhere is rarely reported. We present a case of advanced NPC presenting with generalised LN enlargement involving the neck, axilla, groin, thorax and abdomen, suggestive of lymphoma during the initial presentation. 

## Case Report

A 44-year-old male with underlying chronic rhinosinusitis for 20 years presented with painless bilateral neck swellings for 8 months and bilateral axilla and inguinal LNs swelling for 2 months. The swellings were gradually increasing in size. These were associated with loss of appetite and weight, intermittent fever and lethargy. He had had frequent bilateral nasal blockages, rhinorrhoea with occasional blood-stained mucus, hyposmia and facial congestion for 20 years. The symptoms were temporarily resolved with an oral and topical decongestant. He also complained of bilateral tinnitus for 2 months without other otological symptoms, such as hearing loss, otalgia, otorrhea and vertigo. He had developed lower back pain in the preceding month, which restricted him in work and daily activity. It was associated with bilateral lower limb numbness but no weakness. There were no upper aerodigestive tract obstructive symptoms, such as shortness of breath, noisy breathing or dysphagia. 

He had sought medical attention at other centres, where nasoendoscopy and fine needle aspiration cytology (FNAC) of right neck swelling had been performed. Nasoendoscopy revealed chronic sinusitis features without any obvious mass visible. FNAC results showed the presence of atypical lymphoid cells but could not provide a definitive diagnosis. 

Full blood picture showed normochromic normocytic anaemia (haemoglobin 6.6g/dL), leukocytosis with neutrophilia and monocytosis (white blood cell count 24.4 x 10^9^/L, absolute neutrophil count 20.1 x 10^9^/L, absolute monocyte count 1.7 x 10^9^/L) and pseudothrombocytopenia (platelet count 92 x 10^9^/L). Given that the presentations were more suggestive of lymphoma with generalised lymphadenopathy and the presence of ‘B-symptoms’, computerised tomography (CT) scans of the neck, thorax, abdomen and pelvis were performed for staging purposes. 

**Fig 1:**
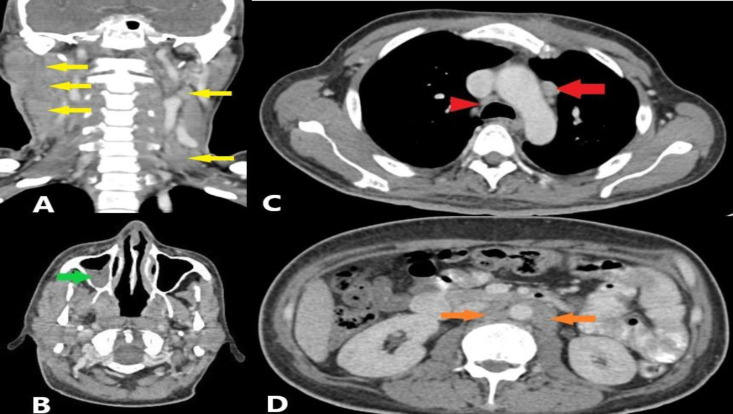
Contrast-enhanced CT scan of neck, thorax, and abdomen. A coronal image of the neck shows bilateral cervical nodes at levels II, III and IV (yellow arrow) (Figure A). Axial image at the level of nasopharynx shows no enhancing lesion seen at the nasopharynx or FOR region but the presence of air-fluid level in the right maxillary sinus secondary to chronic rhinosinusitis (green arrow) (Figure B). Axial image of the thorax depicts the right paratracheal (red arrowhead) and para-aortic LN (red arrow) (Figure C). Axial image at abdominal region shows para-aortic and aortocaval LN enlargement (orange arrow) (Figure D)

The CT neck showed multiple enlarged cervical LNs at level Ia, bilateral level Ib, right level II and III and left level V (Figure 1A). The largest LN was at the right level II, measuring 3.7 cm x 2.1 cm x 5.0 cm, appearing matted and with a necrotic centre. There was a presence of air-fluid level in the right maxillary and sphenoid sinuses. No enhancing lesion was seen in the nasopharynx (Figure 1B), oropharynx or laryngopharynx. Major salivary and thyroid glands appeared normal. Multiple enlarged LNs were also seen at the paratracheal and para-aortic regions on the thorax CT, without any suspicious lung nodules or mass (Figure 1C). Abdomen and pelvis CTs also revealed significant findings with hepatomegaly and multiple ill-defined hypodense lesions at segments III, V and VII. In addition, multiple enlarged LNs were seen in the para-aortic and aortocaval regions (Figure 1D). Overall, imaging features were suggestive of lymphoma Ann Arbor stage IV. 

**Fig 2:**
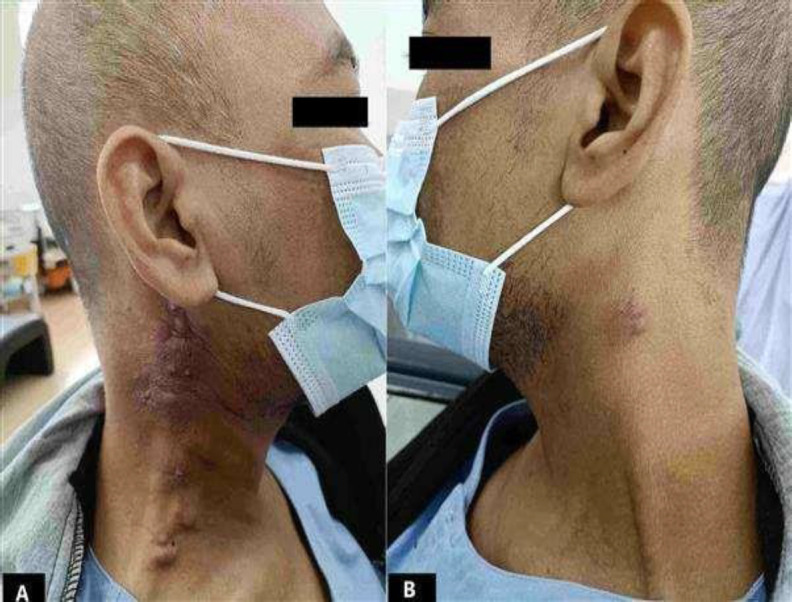
Multiple enlarged LN at right level II, III, IV and V (Figure A) and left level III of the neck (Figure B).

The patient was admitted to the haematology ward due to worsening lethargy and was subsequently referred to the otorhinolaryngology team for excision biopsy for definitive tissue diagnosis. Examination revealed the presence of multiple enlarged LNs at bilateral sides of the neck, namely right level II, III, IV and V (Figure 2A) and left level III (Figure 2B). 

The LN at right level II appeared matted, firm in consistency, immobile and attached to overlying skin. The other LNs were also firm in consistency but mobile and not attached to the overlying skin. In addition to the cervical LNs, there were also multiple enlarged LNs in the bilateral axilla and inguinal regions. Nasoendoscopy revealed mucopus at the bilateral middle meatus and nasopharynx regions, without any obvious mass seen, particularly at the FOR. Another ear, nose and throat examination showed unremarkable findings, and all cranial nerves appeared intact. Examination of the abdomen revealed hepatomegaly, 2 cm below the costal margin, with a smooth surface. 

**Fig 3:**
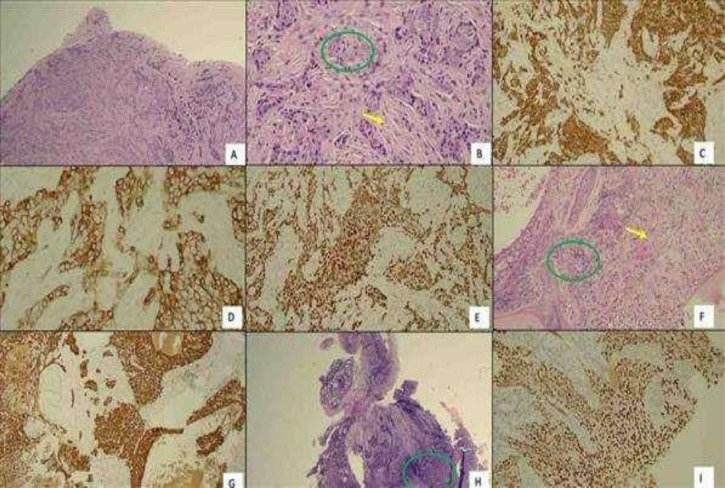
Figure A to E are HPE of excisional biopsy of right cervical LN show tumour infiltration in solid sheets (A, H&E 40x) with a closed up (B, H&E x400) showing the neoplastic cells have oval to round vesicular nuclei (green circle) and desmoplastic changes in the stroma (yellow arrow). The tumour cells were positive to CK AE1 and AE3 (C, x200), CK5/6 (D, x200) and p63 (E, x200). Figure F to G is trephine biopsy cytology showing metastatic carcinoma in green circle (F, H&E x20). There is also evidence of fibrotic/desmoplastic changes of the marrow stroma (yellow arrow) with suppression of other haematological components. Here, the tumour cells have similar morphology as observed in the LN with positive CK AE1 and AE3 (G, 200x). Figure H to I are HPE of biopsy tissue from the nasopharynx mass, extensive crushed artefacts but similar tumour cells were present in green circle (H, H&E 40x) and were positive to p63 (I, 200x)

 Subsequently, the patient was subjected to an excision biopsy of the right level V cervical LN under local anaesthesia. Surprisingly, the histopathology examination (HPE) result showed metastatic carcinoma that was arranged in sheets formed by moderately pleomorphic round-to-oval vesicular nuclei with large nucleoli and eosinophilic cytoplasm. At the periphery of the tumour, clusters revealed many reactive lymphocytes. The surrounding stroma showed desmoplastic reactions. These tumour cells were immunoreactive to cytokeratin (CK) AE1/AE3, CK 5/6, CK 19, epithelial membrane antigen, P63 and CD 117 (Figure 3A to E). The positivity of these markers indicated that the tumour cells were of squamous carcinoma in origin and not of lymphoid cell phenotype. Other immunohistochemical stains were negative, including CK20, CK7, TTF1, p16 and synaptophysin. These findings could suggest that the primary tumour originated from either the head or neck region rather than the lung or thyroid. Despite the absence of Epstein-Barr virus (EBV) markers in the centre, NPC was high in the list of differential diagnoses based on the morphology and immunohistochemistry features. A bone marrow trephine biopsy was performed on the same day and showed metastasis of the primary tumour into the marrow (Figure 3F and G). 

**Fig 4:**
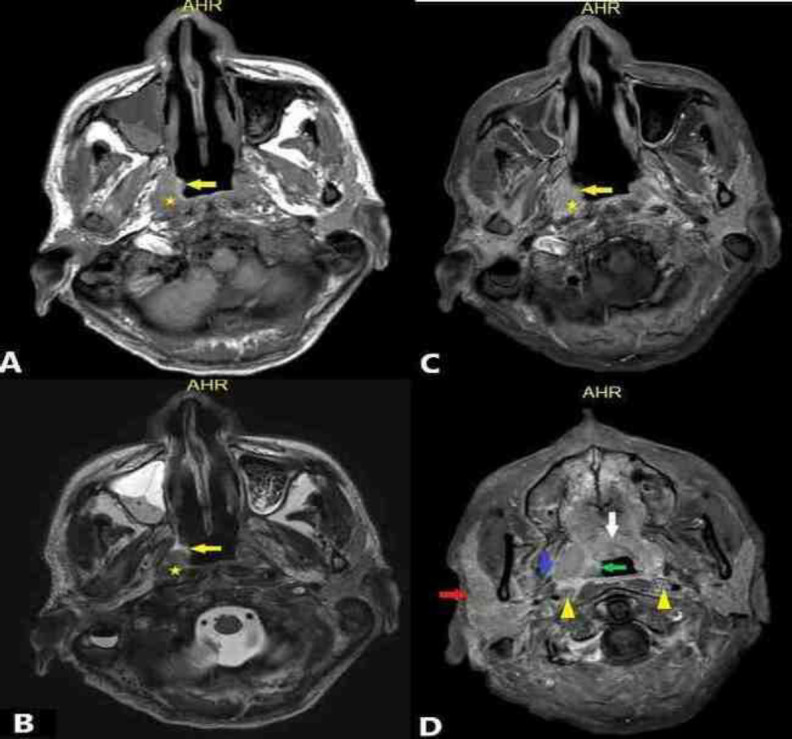
MRI neck in axial T1-weighted (A), T2-weighted (B) and post-contrast (C and D). Figure A, B and C show right nasopharyngeal lesion (yellow arrow) and obliteration of right FOR (star). Figure D shows the extension of the lesion to the right oropharynx (green arrow), right parapharyngeal (blue arrow), soft palate (white arrow) and right parotid (red arrow). Bilateral retropharyngeal LN enlargement is also seen in Figure D (yellow arrowhead)

A multidisciplinary discussion was conducted to discuss the next management option for the patient. Subsequently, magnetic resonance imaging (MRI) of the base of the skull up to the upper thorax was performed to identify the origin of the disease. The MRI showed abnormal signal intensity at the right nasopharynx with obliteration of the right FOR (Figure 4A to C). The lesion extended anteriorly to the soft palate, posteriorly abutting prevertebral muscle, laterally to tensor and palatini muscles, superiorly to the foramen ovale, inferiorly to oropharyngeal mucosa and posterolaterally to the carotid space, with involvement of superficial and deep lobes of the parotid (Figure 4D).

**Fig 5:**
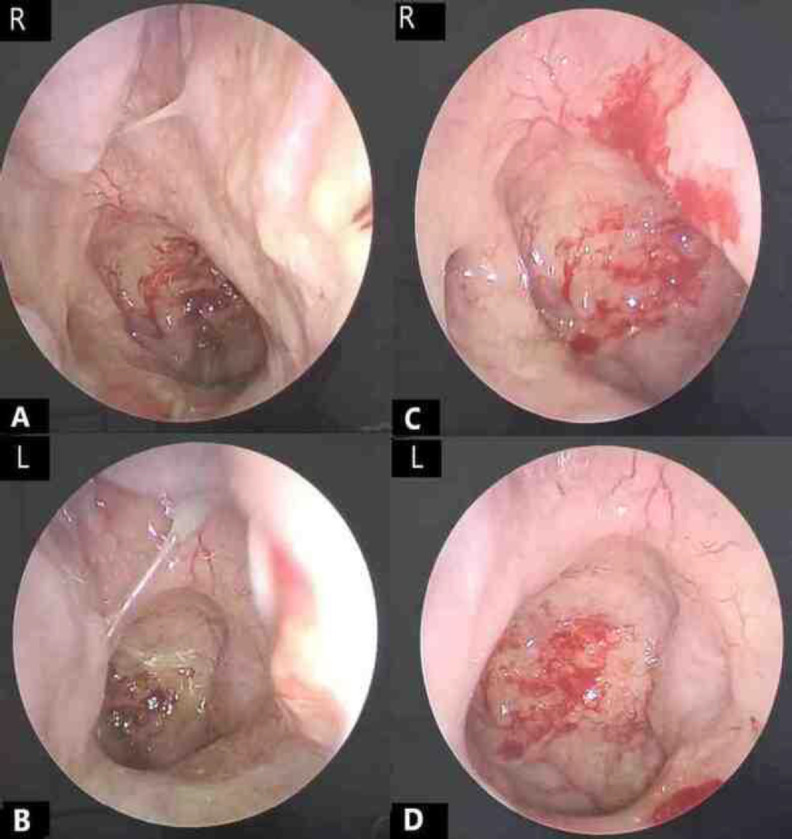
Nasoendoscopy of bilateral nasal cavities before the nasal toilet shows mucopus at the bilateral middle meatus, over the floor of nasal cavities, the pharyngeal opening of the eustachian tube and nasopharyngeal region (mixed with a blood clot), without obvious mass seen (Figure A and B). Nasoendoscopy after the nasal toilet shows inflammation of the nasal and nasopharyngeal mucosa with suspicious irregular mucosa at the centre of the nasopharynx (Figure C and D). R: right, L: left

In view of the positive MRI findings, enhanced nasoendoscopy was repeated using the Storz Professional Image Enhancement System (SPIES), revealing suspicious irregular mucosa at the centre of the nasopharynx (Figure 5). A targeted biopsy was taken and confirmed as non-keratinising differentiated nasopharyngeal carcinoma (Figure 3 H and I). The patient was planned for palliative chemotherapy due to the advanced disease (stage IVB).

## Discussion

 NPC is considered a rare cancer globally, especially in western countries, but is highly prevalent in endemic areas like East and Southeast Asia (1-3). More than 70% of new cases reported in 2018 were from East and Southeast Asia (2). NPC is listed as number nine among cancer incidences and number eight among cancer deaths in Southeast Asia (3). Genetic susceptibility, EBV infection, smoking and consumption of preserved food or salted fish are well-known risk factors (1,3). The most common presentation reported in many studies is neck swelling (1,4,5). A meta-analysis showed that as high as 85% of NPC patients presented with cervical lymphadenopathy, with level II being the most common site, followed by levels III, V and IV. In contrast, levels IA, IB, VI and supraclavicular regions are rarely involved (6). The retropharyngeal node is identified as the first encephalon node and usually metastasis following an orderly pattern of cervical LN, while skip metastasis occurs in around 0.5–7.9% (6). The involvement of this regional LN signifies that the patient is already at least at stage II during the first consultation. Generalised involvement of LNs, such as those in the axilla, groin, thorax and abdomen, are very rarely reported. A retrospective study of 85 cases of axillary LN metastases from a non-mammary primary site showed that 8% of the cases originated from the head and neck, but none from NPC (7). Another case report described a patient with axillary LN metastasis from NPC, but it was a recurrent case (8). To the best of our knowledge, a further inferior group of LNs, especially in the inguinal region, was very unlikely to be associated with NPC. In contrast, these features are ubiquitous in haematological malignancy. Thus, lymphoma was strongly suspected initially in the present case. The early detection of NPC is difficult due to its hidden location, which is impossible to visualise without nasoendoscopy. The most common site of origin of NPC is a pharyngeal recess, also known as the FOR (2,9). 

In addition, early signs and symptoms of NPC are not specific to the disease per se. As seen in our case, a small nasopharyngeal mass may be missed during the first nasoendoscopy if the tumour is submucosally located or obscured by secretion. Our patient had underlying chronic rhinosinusitis for 20 years, which may mask the nasal symptoms of NPC. Furthermore, with the absence of mass at the FOR and secretion and inflammation at the nasopharynx without conspicuous proliferative or ulcerative mass, NPC was not suspected during the initial assessment. Narrow band imaging and enhanced endoscopy, such as the SPIES, are promising tools for early detection of head and neck cancer, including NPC (10). The characteristics of the vascular pattern and appearance of the capillary loop seen during the SPIES endoscopy signify malignancy. This allows for a targeted tissue biopsy for accurate histopathological examination. 

FNAC is always the first investigation of choice in patients with unresolved cervical lymphadenopathy when head and neck malignancy is suspected. 

The reason is to avoid the risk of tumour seeding to the skin in head and neck squamous cell carcinoma, which may upstage the cancer and result in a poor prognosis. In the case of an inadequate sample or inconclusive result, the subsequent investigation of choice is ultrasound-guided FNAC. The first FNAC of the present case was inconclusive but showed some atypical lymphoid cells. Other investigations, such as CT scans, were suggestive of lymphoma or advanced cancer. Thus, we immediately performed an excisional biopsy to avoid delays in diagnosis and treatment. In addition, tumour seeding was no longer a concern in this case because the patient was already at an advanced stage. Unfortunately, the HPE of the excision biopsy revealed metastatic carcinoma, which could have originated from the head and neck, lung or thymus. Special stains for NPC, such as EBV-encoded nonpolyadenylated RNAs (EBERs), EBV nuclear antigen 1 (EBNA1) and latent membrane proteins 1 and 2 (LMP1 and 2), are currently found to be helpful (11). Unfortunately, they were not available at our centre at that time. The case became more complex because the primary tumour could not be identified at that stage. 

 MRI is an excellent investigation in the case of submucosal NPC because it can delineate soft tissue better than a CT scan (2), as shown in the present case. Advanced metastasis with very minimal findings on nasoendoscopy made this case very challenging to diagnose. With the help of the MRI and nasopharyngeal biopsy, NPC was finally concluded as a diagnosis, but the patient was already in an advanced stage. Long-term survival for advanced disease was still poor even if the tumour was sensitive to radiotherapy and chemotherapy (12).

 Traditionally, single modality treatment with radiotherapy is the primary treatment for early-stage NPC (stages II and I), while the combination of radiotherapy and chemotherapy is reserved for the later stages (stages III and IV). The management strategies have changed because many studies have been conducted and have shown better overall survival outcomes with the new treatment regime. Concurrent radiotherapy and chemotherapy have become a better choice for stage II disease, while induction chemotherapy followed by concurrent radiotherapy and chemotherapy are preferred for late-stage disease (13). 

Our patient was planned for palliative chemotherapy because of the advanced disease, but unfortunately his Eastern cooperative oncology group performance status was poor, and he developed sepsis secondary to community-acquired pneumonia. His condition deteriorated before starting chemotherapy and he passed away about five months after the first presentation. 

## Conclusion

NPC has an excellent response to radiotherapy and chemotherapy in an early stage, with a good prognosis compared to the late stage. Delayed diagnosis may be caused by the deep location of the nasopharynx, association with other sinonasal pathology such as rhinosinusitis that masks nasal symptoms or signs of NPC, and submucosal NPC with generalised lymphadenopathy without obvious nasopharyngeal mass that may be more suggestive of a malignant lymphoproliferative disorder. Thorough ear, nose and throat examinations should be carried out in all patients presenting with cervical lymphadenopathy and NPC should be top of the list, especially in patients from East or Southeast Asia. Narrow band imaging or enhanced endoscopy with the SPIES should be performed if available, especially at the suspicious lesion. CT scans are readily available in most centres and are usually the investigation of choice in most NPC cases. MRI has better soft tissue delineation but is not available in many centres with long waiting lists. It should be arranged without delay in selected cases with highly suspicious NPC but negative CT scan findings. 
